# Cognitive–behavioural therapy in medication-treated adults with attention-deficit/hyperactivity disorder and co-morbid psychopathology: a randomized controlled trial using multi-level analysis

**DOI:** 10.1017/S0033291715000756

**Published:** 2015-05-29

**Authors:** S. Young, M. Khondoker, B. Emilsson, J. F. Sigurdsson, F. Philipp-Wiegmann, G. Baldursson, H. Olafsdottir, G. Gudjonsson

**Affiliations:** 1 Division of Brain Sciences, Department of Medicine, Centre for Mental Health, Imperial College London, London, UK; 2 Broadmoor Hospital, West London Mental Health NHS Trust, Crowthorne, UK; 3 Reykjavik University, Reykjavik, Iceland; 4 King's College London, Institute of Psychiatry, Psychology and Neuroscience, London, UK; 5 Department of Applied Health Research, University College London, London, UK; 6 Landspitali – The National University Hospital of Iceland, Reykjavik, Iceland; 7 University of Iceland, Reykjavik, Iceland

**Keywords:** Attention-deficit/hyperactivity disorder, cognitive–behavioural therapy, randomized controlled trials, reasoning and rehabilitation, R & R2, treatment

## Abstract

**Background:**

Attention-deficit/hyperactivity disorder (ADHD) is a neurodevelopmental disorder characterized by high rates of co-morbid psychopathology. Randomized controlled trials of multimodal interventions, combining pharmacological and psychological treatments, have shown a robust treatment effect for ADHD symptoms but outcomes for co-morbid symptoms have been mixed. This may be accounted for by the type of intervention selected and/or by methodological problems including lack of follow-up and low power. The current study addressed these limitations in a parallel-group randomized controlled trial conducted in Iceland.

**Method:**

A total of 95 adult ADHD patients who were already being treated with medication (MED) were randomly assigned to receive treatment as usual (TAU/MED) or 15 sessions of cognitive–behavioural therapy (CBT/MED) using the *R&R2ADHD* intervention which employs both group and individual modalities. Primary measures of ADHD symptoms and severity of illness, and secondary measures of anxiety, depression and quality of life were given at baseline, end of treatment and 3-month follow-up. Primary outcomes were rated by clinicians blind to treatment condition assignment.

**Results:**

CBT/MED showed overall (combined outcome at end of treatment and 3-month follow-up) significantly greater reduction in primary outcomes for clinician-rated and self-rated ADHD symptoms. Treatment effect of primary outcomes was maintained at follow-up, which suggests robust and lasting findings. In contrast to the primary outcomes, the secondary outcomes showed significant improvement over time.

**Conclusions:**

The study provides evidence for the effectiveness of *R&R2ADHD* and demonstrates that there are differential effects over time for ADHD symptoms *versus* co-morbid problems, the latter taking longer to show positive effects.

## Introduction

Attention-deficit/hyperactivity disorder (ADHD) is a common neurodevelopmental disorder characterized by core symptoms of inattention, hyperactivity and/or impulsivity and associated with significant social, educational and occupational impairments. Longitudinal and epidemiological studies have shown ADHD to be a chronic life-long condition emerging in childhood (Biederman & Faraone, 2005; Sobanski *et al.* 2007; Young & Amarasinghe, 2010; Guldberg-Kjär *et al.* 2013). Meta-analytic reviews have estimated the prevalence of ADHD in adulthood to be 2.5–5% (Simon *et al.* 2009; Willcutt, 2012). It is a complex disorder characterized by high rates of co-morbidity including mood disorders, anxiety, alcohol and drug abuse, and interpersonal relationship problems (Pliszka, 1998; Shaw *et al.* 2012). Indeed cross-sectional, retrospective and follow-up studies have indicated that ADHD patients are up to 80% more likely to develop other psychiatric difficulties (Sobanski *et al.* 2007; Barkley *et al.* 2008). A large, nationally representative US sample demonstrated that ADHD had an increased lifetime prevalence of all psychiatric disorders, even after adjusting for sociodemographic characteristics (Bernardi *et al.* 2012).

In the UK, pharmacological interventions are the first-line recommended treatment of ADHD in adults (National Institute for Health and Clinical Excellence, 2009; Bolea-Alamañac *et al.* 2014) and meta-analytic studies have reported a medium to large treatment effect (Mészáros *et al.* 2009). However, up to 50% of medicated adults may not fully respond to medication (Wender, 1998; Wilens *et al.* 2002; Prince, 2006; Safren, 2006). By contrast, psychological treatments have received less attention but generally support that medication significantly augments the outcome of therapy; a systematic review examining the effect of treatment modality on long-term outcomes concluded that the combination of pharmacological and non-pharmacological treatment was most consistently associated with improved long-term outcomes and large effect sizes. Furthermore, the age of treatment initiation and duration of treatment did not markedly affect the proportion of improved outcomes reported (Shaw *et al.* 2012). Only one study has been conducted comparing participants randomized to receive combined cognitive–behavioural therapy (CBT) and medication (dextroamphetamine) *versus* CBT and placebo; this study found ADHD symptom outcomes using CBT alone were as good as when both medication and CBT interventions were combined (Weiss *et al.* 2012). These findings are impressive and have great implications for treatment with CBT as a stand-alone intervention in this population. However the findings need to be replicated.

International guidelines recommend a multimodal treatment approach comprising both pharmacological and psychological interventions (Seixas *et al.* 2012). The common clinical reality, however, is a reliance on medication as first line. This is due to the limited evidence base currently available for psychological intervention for treating adults with ADHD. More research is needed in this area and this study makes an important contribution to the field. When choosing psychological interventions, the selection of group-delivered treatment is preferable as it is resource and cost effective (National Institute for Health and Clinical Excellence, 2009). Randomized controlled trials (RCTs) that evaluate the combination of these treatments (*versus* medication alone) have reported medium to large treatment effects for both self-rated and clinician-rated improvements in ADHD symptoms (Stevenson *et al.* 2002; Safren *et al.* 2005, 2010; Safren, 2006). Furthermore, some treatment effects appear to be robust, with two studies reporting sustained effects 1 year later (Stevenson *et al.* 2002; Safren *et al.* 2010).

There have only been three RCTs involving a group psychological intervention that have evaluated co-morbid psychopathology at outcome (i.e. anxiety and depression). These have reported mixed findings; in contrast to the findings of Emilsson *et al.* (2011), two studies reported no significant improvement in symptoms (Solanto *et al.* 2010; Hirvikoski *et al.* 2011). The weakness with the latter two studies is that there were no follow-up data, which means that any possible reduction in co-morbid symptoms after the end of treatment was not assessed. Emilsson *et al.* (2011) compared medicated patients randomly assigned to either receive medication (MED) and CBT (CBT/MED) or treatment as usual (TAU/MED) and reported large treatment effects at 3-month follow-up for self-rated attention, hyperactivity/impulsivity, anxiety, depression, emotional control, antisocial behaviour and social functioning and clinician ratings of illness severity and ADHD core symptoms. However, there was no significant treatment effect for anxiety and depression at the end of treatment, which is consistent with the Hirvikoski *et al.* (2011) and Solanto *et al.* (2010) studies, and suggests that co-morbid problems may take longer to show positive effect than the core ADHD symptoms. This finding requires further investigation.

However, the Emilsson *et al.* (2011) community study only involved 54 participants, 27 in each group, and there was a substantial amount of missing data at the end of treatment and at 3-month follow-up for both the CBT/MED and TAU/MED groups. The presence of missing data may potentially lead to a biased estimate of the treatment effect, which was not appropriately dealt with in the previous study due to the limitations of the statistical approach taken. For example, there is evidence that age and antisocial personality (ASP) traits are significant predictors of failure to attend follow-up appointments (Gudjonsson *et al.* 2004). A recommended way (White *et al.* 2011) to reduce possible bias is to analyse all the observed outcome data via the maximum likelihood method under the data missing at random (MAR) assumption, which requires the inclusion of any relevant predictors of missing data in the analysis model.

The current study therefore addressed these methodological weaknesses by using a larger sample randomized to either CBT/MED or TAU/MED and including a 3-month follow-up. We performed an intention-to treat analysis using a linear mixed model and analysed for three possible predictors of missing data: gender, age, and ASP traits. Thus we analysed the effects of treatment over time (i.e. end of treatment *versus* at 3-month follow-up) as well as overall group differences in the outcome measures whilst controlling for possible group imbalances caused by missing data. It was hypothesized that the CBT/MED group would show significantly greater reduction in the primary outcomes of ADHD core symptoms and illness severity compared with the TAU/MED group after adjusting for missing data and possible confounders. A similar treatment effect was expected for secondary outcomes of anxiety, depression and quality of life. Treatment gains were expected to be maintained at 3-month follow-up.

## Method

### Trial design

A parallel-group RCT was conducted at an ADHD out-patient setting within the Mental Health Services at Landspitali – The National University Hospital of Iceland. All participants meeting inclusion criteria were independently and individually randomly allocated (1:1) to receive the *R&R2ADHD* programme (CBT/MED) or treatment as usual (TAU/MED). Assessments occurred at three time points: baseline, end of treatment and 3 months after treatment. The study was registered with the International Clinical Trials Registry (no. 12611000533998).

An *a priori* power calculation was conducted using G*Power (Faul *et al.* 2007) to estimate the sample size required. Safren *et al.* (2005) obtained a large effect size between CBT-treated medicated adults with ADHD and those treated by medication only. In view of the fact that the Safren *et al.* study involved individual rather than group therapy, where the effect size is likely to be larger, we used an estimated effect size of 0.50. The power was set at 80% and the level of significance at 0.05. This suggested a sample of *n* = 51 in each group.

### Participants

Participants were either hospital referrals for out-patient rehabilitation made by the Mental Health Services at the Landspitali University Hospital, referrals from private practitioners or self-referrals from an advertisement placed with a national ADHD support group (Icelandic ADHD Association). Participants were eligible for the study if they were over 18 years of age, had a current ADHD diagnosis and had been stable on prescribed ADHD medication for at least 1 month. Participants were asked to keep dosages unchanged during the study. Exclusion criteria were severe mental illness (i.e. psychotic disorders, bipolar disorder), severe eating disorder, active suicidal ideation, active drug abuse and history of intellectual impairment as the treatment programme would not be suitable for these patient groups without modification. Exclusion criteria were assessed from a review of medical records, in addition to a baseline assessment by an experienced mental health practitioner (see Baseline assessments section).

**Fig. 1.**
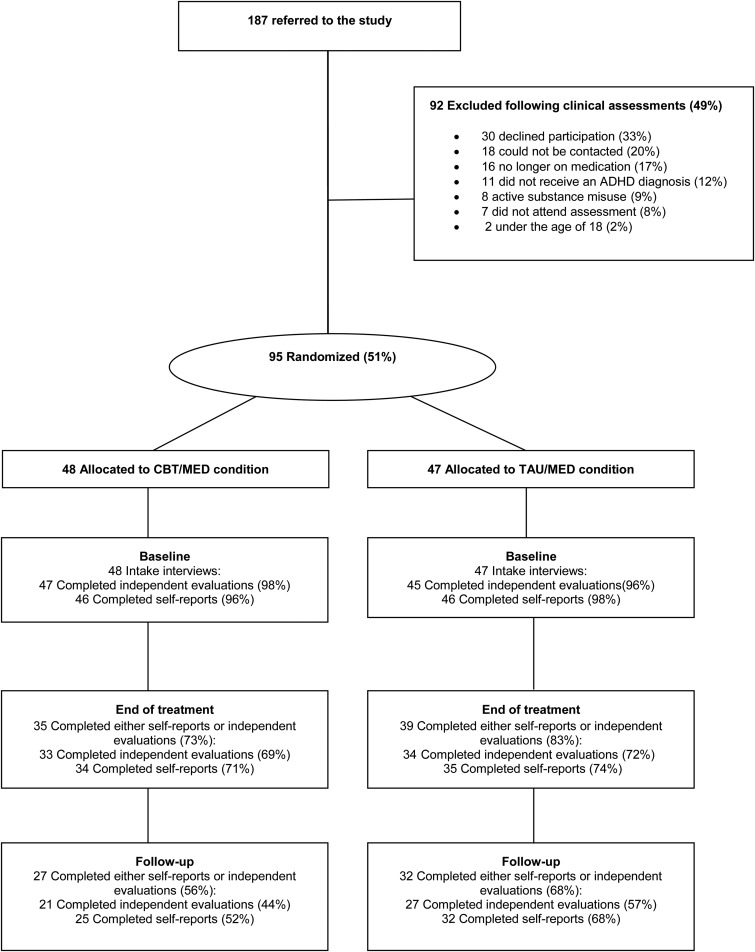
Flowchart of patient participation. ADHD, Attention-deficit/hyperactivity disorder; CBT/MED, cognitive–behavioural therapy plus medication; TAU/MED, treatment as usual plus medication.

Of 187 referrals, 73 (39%) were received from private practice psychiatrists, 56 (30%) were referred by psychiatrists, psychologist or psychiatric nurses at the Mental Health Services at Landspitali University Hospital and 56 (30%) were referred from advertisements to the members of the Icelandic ADHD Association. Out of the 187 referrals, 95 (51%) participants took part in the study. Fig. 1 presents the reasons for non-participation, the most common being declining to participate (*n* = 30), not contactable (*n* = 18) and stopped medication (*n* = 16). Of the participants, 11 were excluded because at the study intake interview (conducted by an experienced mental health practitioner) they were not diagnosed with ADHD [according to Diagnostic and Statistical Manual of Mental Disorders (DSM)-IV criteria]; 62 of the participants were female (mean age = 35.00, s.d. = 11.81 years) and 33 were male (mean age = 35.45, s.d. = 11.62 years).

Demographic and clinical characteristics of the study sample are presented in Table 1. All medication was prescribed by psychiatrists and, at baseline, 79 (83.2%) were taking methylphenidate and 16 (16.8%) atomoxetine. Five participants were also taking bupropion. In addition, 63 (66.3%) participants were taking other prescribed medications (mean number of medications 2.45, s.d. = 1.39) including antidepressants, benzodiazepines, insulin and ibuprofen. There was considerable co-morbidity present as, in addition to ADHD, participants reported co-morbid depression (63.2%), anxiety (36.8%), and history of drug/alcohol abuse (15.8%). Of the participants, seven (7.4%) reported to have been diagnosed with a personality disorder and four with Asperger's syndrome in childhood. Four reported having post-traumatic stress disorder and two with a history of eating disorder.

**Table 1.** Demographic, clinical and baseline characteristics of the study sample (n = 95)

	Total (*n* = 95)	CBT/MED (*n* = 48)	TAU/MED (*n* = 47)	Statistics
Gender, *n* (%)				
Men	33 (43.7)	18 (37.5)	15 (31.9)	*χ*^2^ = 0.327, *p* = 0.57
Women	62 (65.3)	30 (62.5)	32 (68.1)	
Age, years				
Mean	35.17	34.19	36.17	*t*_93_ = 0.826, *p* = 0.41
s.d.	11.68	10.58	12.75	
Range	18–73	18–68	18–73	
Marital status, *n* (%)				
Single	47 (49.5)	23 (48.9)	24 (51.1)	*χ*^2^ = 0.189, *p* = 0.66
In a relationship	47 (49.5)	24 (51.1)	23 (48.9)	
Employment status, *n* (%)				
Employed	41 (43.2)	18 (37.5)	23 (48.9)	*χ*^2^ = 2.236, *p* = 0.33
Training	22 (23.2)	14 (29.2)	8 (17.0)	
Pension/unemployed	32 (33.7)	16 (33.3)	16 (34.0)	
Medical history, *n* (%)				
History of serious illness	27 (28.4)	13 (27.1)	14 (29.8)	*χ*^2^ = 0.085, *p* = 0.77
History of head trauma	36 (37.9)	19 (39.6)	17 (36.2)	*χ*^2^ = 0.118, *p* = 0.73
History of serious accidents	33 (34.7)	17 (35.4)	16 (34.0)	*χ*^2^ = 0.020, *p* = 0.89
History of receiving psychotherapy	69 (72.6)	33 (68.8)	36 (76.6)	*χ*^2^ = 0.735, *p* = 0.39
ADHD-specific medication, *n* (%)				
Methylphenidate	73 (83.2)	40 (83.3)	33 (83.0)	*χ*^2^ = 0.002, *p* = 0.963
Atomoxetine	16 (16.8)	8 (16.7)	8 (17.0)	*χ*^2^ = 0.002, *p* = 0.963
Bupropion	5 (5.3)	3 (6.3)	2 (4.3)	*χ*^2^ = 0.234, *p* = 0.629
Other medications^a^	63 (66.3)	32 (66.7)	31 (66.0)	*χ*^2^ = 0.005, *p* = 0.942

CBT/MED, Cognitive–behavioural therapy plus medication; TAU/MED, treatment as usual plus medication; s.d., standard deviation; ADHD, attention-deficit/hyperactivity disorder.

a Other medications include, for example, antidepressants, benzodiazepines, insulin, ibuprofen and various other medications.

### Interventions

*R&R2ADHD* is a CBT intervention programme developed for youth and adults with ADHD (Young & Ross, 2007). It is a revised version of the 35-session Reasoning & Rehabilitation prosocial competence training programme which has a strong evidence base (Tong & Farrington, 2006). It was revised to be a shorter and more relevant intervention for individuals presenting with symptoms associated with ADHD. The revision, *R&R2ADHD*, is a structured, manualized programme consisting of 15 sessions of 90 min (excluding a mid-session break) and aims to decrease ADHD symptoms and improve social, problem-solving and organizational skills. It has five treatment modules: (a) neurocognitive, e.g. learning strategies to improve attentional control, memory, impulse control and planning; (b) problem solving, e.g. developing skilled thinking, problem identification, consequential thinking, managing conflict and making choices; (c) emotional control, e.g. managing feelings of anger and anxiety; (d) prosocial skills, e.g. recognition of the thoughts and feelings of others, empathy, negotiation skills, and conflict resolution; and (e) critical reasoning, e.g. evaluating options and effective behavioural skills. *R&R2ADHD* is a group treatment supplemented by one-to-one meetings with a mentor. In the present study the group sessions were delivered twice per week. The mentors met with the participants between each group session in order to support participants to transfer skills learned in the group into their daily lives. Programme integrity was ensured by group sessions being delivered according to the manual by experienced CBT therapists, who had received training and accreditation to deliver the programme. The mentoring sessions were provided by psychology students who also received training, supervision and written guidance.

Treatment completion was classified as ≥12 sessions, representing 80% attendance of the programme.

TAU was classified as receiving usual treatment, which included both pharmacological and non-pharmacological treatments.

### Measures

#### Baseline assessments

All referrals who could be contacted and who consented to participate in the study were interviewed prior to randomization to ascertain clinical diagnosis according to DSM-IV criteria using the MINI International Neuropsychiatric Interview (Sheehan *et al.* 1998) by an experienced mental health practitioner. In addition, the 54-item Gough Socialization Scale (Gough, 1960) was used to measure ASP traits because these have been found to be associated with failure to attend follow-up appointments after treatment in an Icelandic sample (Gudjonsson *et al.* 2004). In addition, Young & Gudjonsson (2006) found that community patients diagnosed with ADHD commonly had ASP traits as measured by the Gough Socialization Scale, which might be related to missing follow-up data (dropouts). There may of course be other possible confounding variables not included in the analysis, such as substance misuse, which may act similarly to APD traits.

Sociodemographic data and medical information from a review of clinical records were obtained (see Table 1). In addition, a battery of psychometric tests assessed the primary (ADHD core symptoms and illness severity) and secondary outcomes (anxiety, depression, quality of life) at baseline, at the end of treatment and at 3-month follow-up as follows.

#### Primary outcomes

The Kiddie-Schedule for Affective Disorders and Schizophrenia (K-SADS), ADHD section, is a measure of symptom change and severity of ADHD symptomatology (Kaufman *et al.* 1996). The 18-item questionnaire was completed by clinicians who were blind to treatment allocation. Magnússon *et al.* (2006) have reported that the scale has good reliability and validity in an Icelandic sample.

The Clinical Global Impression (CGI; National Institute of Mental Health, 1985) is a single-question observer rating of severity of illness on a seven-point scale. It is based on judgment regarding impairment in functioning, symptom severity and distress or coping and is supported by examples of these factors. It was completed by clinicians who were blind to treatment condition. The CGI has been widely used in treatment evaluation studies and has been found to correlate with ADHD severity measured by the adult ADHD Investigator Symptom Rating Scale (Spencer *et al.* 2010).

The Barkley Current Symptoms Scale (Barkley, 1998) is an 18-item self-report questionnaire that measures ADHD symptoms and corresponds with the DSM-IV criteria (APA, 1994). The questionnaire consists of a total scale made up from two subscales; one measures inattention and the other hyperactivity/impulsivity. Magnússon *et al.* (2006) found a high correlation between informants’ ratings of symptoms and interview-based diagnoses in childhood and adulthood in an Icelandic sample. The correlations were 0.49 (males) and 0.58 (females) for childhood symptoms and 0.50 (males) and 0.55 (females) for symptoms in adulthood.

#### Secondary outcomes

The Beck Anxiety Inventory (BAI; Beck & Steer, 1993) is a 21-item self-report questionnaire that is widely used in clinical practice and research.

The Beck Depression Inventory (BDI; Beck *et al.* 1961) is a 21-item self-report questionnaire to assess symptoms and severity of depression.

The Quality of Life Scale (QOLS; Flanagan, 1978, 1982) is a 16-item scale that assesses attitudes toward a person's own quality of life. The QOLS was originally developed for research in healthy populations, but it has been used in several international studies with chronic illnesses (Burckhardt & Anderson, 2003) and among cancer patients in Iceland (Friðriksdóttir *et al.* 2011). Burckhardt & Anderson (2003) have demonstrated good construct validity of the scale in a sample of chronically ill and healthy adults from American and Swedish databases.

### Procedure

All referrals who could be contacted and who agreed to participate in the study were assessed for eligibility by participating in an interview to confirm their ADHD diagnosis, and assess co-morbidity. A battery of self-rated and clinician-rated evaluations (the latter being blind to treatment allocation) was conducted at the three time points (baseline, post-treatment and at 3-month follow-up). Participants were randomized to either the CBT/MED or TAU/MED condition. Randomization was conducted independently by a psychiatrist at Landspitali University Hospital, who was not involved in the study. The psychiatrist had no information about the participants and received numbers that were pre-assigned to the participants. Block randomization by using equal block sizes was performed at the time of each study phase. Only the final randomization numbers were reported back to the researchers to protect the concealment of the allocation as proposed in various studies (Beller *et al.* 2002; Schulz & Grimes, 2002). The *R&R2ADHD* programme was delivered twice per week by experienced CBT therapists who had attended training to deliver the programme. Group participants met their mentor between group sessions for at least 30 min. Mentors attended a training session to fulfil this role involving an introduction to the programme and the mentoring role. In addition, mentors have a manual that guides them through topics to be discussed within the mentoring session. They received supervision once a fortnight from the lead group therapist. There were five *R&R2ADHD* treatment groups in total. Participants in the TAU/MED condition received pharmacological intervention and other non-pharmacological interventions but these were not systematically provided or recorded.

### Statistical analyses

The statistical analysis involved two sequential steps. First, we applied a logistic regression model to identify factors (i.e. gender, age and ASP traits) that might predict the probability of missing data. Only age was found to be significantly associated with the probability of dropouts (i.e. younger participants in both groups more often failed to attend the assessment interviews with the independent raters: K-SADS, *Z* = −2.19, *p* = 0.029; CGI, *Z* = −2.35, *p* = 0.019).

Second, an intention-to-treat analysis (individuals analysed in the group to which they were randomized) of available outcome data was performed to estimate the effect of offering the treatment using a linear mixed model. In view of the significant relationship found between age and missing data dropouts, we controlled for age in the linear mixed model. The random component of the mixed model included a random intercept term for subject identifier to take account of between-subject variability and the correlation between the repeated measures. Within the fixed part of the model, the treatment effect was adjusted for time (a binary indicator of whether an outcome measure corresponds to follow-up or end of treatment) and the baseline measures of the respective outcome in all models. We tested condition × time interactions, but none was found statistically significant and therefore these were excluded from the model.

The amount of missing data at baseline was minimal, but there was a substantial proportion (leading up to 50% by the follow-up) of missing data in the completion of the outcome measures due to study dropouts. The dropout rate was similar for both the CBT/MED and TAU/MED groups (see Fig. 1), with *χ*^2^ tests revealing no differences between groups.

Two of the commonly recommended approaches for dealing with the risk of potential bias due to missing data are multiple imputation and complete case analysis via maximum likelihood. Multiple imputation is appropriate when there are missing data in covariates which is not the case in our study. When missing data occur only in the outcome variables then complete case analysis via maximum likelihood produces unbiased results, provided that the variables associated with the outcome being missing are included as covariates (under a MAR assumption). We adopted the complete case analysis approach under a MAR assumption as we had missing data only in the outcome variables. Covariates driving missingness were identified using a logistic regression analysis and an analysis of all observed outcome data was performed using a linear mixed model via maximum likelihood method controlling for predictors of missing data, which should produce unbiased estimates under a MAR assumption (White *et al.* 2011).

Adjusted effect sizes (Cohen's *d*) were obtained by calculating the residuals from the respective linear mixed model with the condition term excluded, and then calculating the standardized mean difference of the adjusted outcome (residuals) between groups. The calculation was conducted using the user contributed STATA module COHEND (http://ideas.repec.org/c/boc/bocode/s457235.html), which calculates effect sizes by adjusting for uneven group sizes.

Descriptive characteristics of the demographic and clinical sample data as well as the outcome measures are presented as means and standard deviations. To assess differences between the CBT/MED and TAU/MED conditions at baseline, independent-sample *t* tests were performed and *χ*^2^ tests were used to analyse categories and categorical data, respectively.

### Ethical standards

All procedures contributing to this work comply with the ethical standards of the relevant national and institutional committees on human experimentation and with the Helsinki Declaration of 1975, as revised in 2008.

## Results

### Baseline characteristics

There were no significant differences between the CBT/MED and the TAU/MED groups in the demographic background data (see Table 1). As far as the baseline outcome measures are concerned, there were no significant differences between groups with the exception that the TAU group had a lower Barkley Current Symptoms Scale hyperactivity/impulsivity score compared with the CBT group (Table 2).

**Table 2.** Outcome measures in the CBT/MED and TAU/MED conditions and statistics of the baseline measurements

Outcome	CBT/MED			TAU/MED			Statistics of the baseline measurements between groups
	Baseline	End of treatment	Follow-up	Baseline	End of treatment	Follow-up	
K-SADS total	39.96 (5.71)	30.60 (7.33)	30.95 (5.53)	37.53 (8.05)	35.47 (6.40)	35.67 (5.86)	*t*_79_ = 1.253, *p* = 0.214
*n*	47	33	21	45	34	27	
K-SADS inattention	21.70 (3.54)	16.55 (3.98)	17.43 (3.60)	20.87 (4.05)	19.88 (3.94)	19.26 (3.49)	*t*_90_ = 1.054, *p* = 0.296
*n*	47	33	21	45	34	27	
K-SADS hyperactivity/impulsivity	17.66 (3.76)	14.06 (4.45)	13.52 (3.86)	16.67 (4.78)	15.58 (4.15)	16.41 (3.93)	*t*_90_ = 1.110; *p* = 0.270
*n*	47	33	21	45	34	27	
CGI	3.96 (0.81)	3.03 (1.05)	3.14 (0.79)	3.91 (1.10)	3.79 (0.77)	3.80 (0.96)	*t*_90_ = 0.231, *p* = 0.818
*n*	47	33	21	45	34	27	
BCS combined	28.80 (9.26)	17.26 (7.58)	14.72 (8.31)	25.15 (11.09)	21.57 (9.75)	22.34 (9.17)	*t*_90_ = 1.714, *p* = 0.090
*n*	46	34	25	46	35	32	
BCS inattention	16.28 (5.49)	10.59 (4.40)	9.60 (5.34)	15.58 (6.57)	13.71 (5.72)	14.19 (5.85)	*t*_90_ = 0.637, *p* = 0.526
*n*	46	34	25	46	35	32	
BCS hyperactivity/impulsivity	12.52 (5.16)	6.68 (5.01)	5.12 (4.05)	9.63 (5.97)	7.86 (5.92)	8.16 (5.13)	*t*_90_ = 2.448, *p* = 0.016
*n*	46	34	25	46	35	32	
BAI anxiety	13.84 (8.69)	10.41 (9.10)	6.29 (4.53)	14.60 (7.43)	14.37 (10.67)	12.13 (7.65)	*t*_87_ = −0.443, *p* = 0.659
*n*	44	34	24	45	35	31	
BDI depression	12.72 (8.62)	8.38 (6.99)	5.04 (5.60)	16.09 (10.23)	14.00 (10.45)	13.14 (7.99)	*t*_87_ = −1.690, *p* = 0.095
*n*	46	34	24	43	34	29	
Quality of life	71.38 (14.40)	74.50 (14.53)	79.84 (11.07)	69.54 (14.91)	70.94 (16.29)	72.22 (14.31)	*t*_89_ = 0.595, *p* = 0.553
*n*	45	34	25	46	35	32	

Data are given as mean (standard deviation).

CBT/MED, Cognitive–behavioural therapy plus medication; TAU/MED, treatment as usual plus medication; K-SADS, Kiddie-Schedule for Affective Disorders and Schizophrenia, attention-deficit/hyperactivity disorder section; CGI, Clinical Global Impression; BCS, Barkley Current Symptoms Scale; BAI, Beck Anxiety Inventory; BDI, Beck Depression Inventory.

### Programme completion

Just over half of the participants completed the programme (*n* = 25; 52.1%).

### Outcomes

In Table 3 a selected output from the linear mixed model analyses is given. Each row shows the coefficient of the treatment indicator (0 = TAU/MED, 1 = CBT/MED) and the relevant inferential statistics of the named outcome. All models included a random intercept term for subject identification and controlled for age, time (indicator of whether the measurement corresponds to end of treatment or follow-up or end of treatment) and the baseline measurement differences of each respective outcome variable. Estimates of the adjusted overall mean differences (i.e. combining the scores from end of treatment and at 3-month follow-up) between the CBT/MED and TAU/MED groups and the corresponding *p* values are provided. There was a significant main effect for all the outcome measures except for the BAI and quality of life measures. The group difference on the BAI was very close to being statistically significant at the 5% level, which suggests that the CBT group tended to have a considerably reduced BAI scores compared with the TAU group.

**Table 3.** Estimated treatment effect from the linear mixed-model analyses with adjusted effect sizes (Cohen's d) from the model

Outcome	*β* Coefficient	s.e.	*p*	95% CI	Effect size: *d*
K-SADS total	−5.41	1.03	<0.001	−7.43 to −3.38	0.65
K-SADS inattention	−3.22	0.70	<0.001	−4.6 to −1.84	0.56
K-SADS	−2.11	0.60	<0.001	−3.29 to −0.93	0.51
hyperactivity/impulsivity					
CGI	−0.79	0.17	<0.001	−1.12 to −0.46	0.64
BCS combined	−6.60	1.33	<0.001	−9.19 to −3.99	0.46
BCS inattention	−3.63	0.80	<0.001	−5.21 to −2.06	0.42
BCS	−3.10	0.73	<0.001	−4.50 to −1.63	0.46
hyperactivity/impulsivity					
BAI anxiety	−3.11	1.72	0.071	−6.49 to 0.26	0.21
BDI depression	−4.84	1.51	0.001	−7.79 to −1.89	0.32
Quality of life	3.36	2.50	0.180	−1.55 to 8.26	0.14

s.e., Standard error; CI, confidence interval; K-SADS, Kiddie-Schedule for Affective Disorders and Schizophrenia, attention-deficit/hyperactivity disorder section; CGI, Clinical Global Impression; BCS, Barkley Current Symptoms Scale; BAI, Beck Anxiety Inventory; BDI, Beck Depression Inventory.

There was an overall effect of time (end of treatment *versus* 3-month follow-up) adjusted for baseline, group and age, on the three secondary measures: BAI (*Z* = −2.53, *p* = 0.011, *d* = 0.58), BDI (*Z* = −2.2, *p* = 0.025, *d* = 0.52) and quality of life (*Z* = 2.47, *p* = 0.014, *d* = 0.56), showing steady improvement over time in the treatment group. No significant time change was noted regarding the primary outcome measures.

## Discussion

This was a randomized controlled (intention-to-treat analysis) study of the effectiveness of CBT (*R&R2ADHD*) in medication-treated adults with ADHD. The study employed a sophisticated analysis (using a linear mixed model) to control for confounders associated with missing data, between-subject variability, and the correlation between the measures over time. The current study thus provides a more rigorous methodology and robust statistical analysis of outcome than used in previous research. In the current study, the treatment effect in the model was adjusted for time, which allowed us to investigate whether or not the treatment effectiveness noted at the end of treatment was maintained or improved at 3 months follow-up, in addition to investigating the overall effect of the end of treatment and 3-month follow-up combined.

The results revealed several robust findings. As hypothesized, the CBT/MED group showed overall (combined outcome at post-treatment and follow-up) significantly greater reduction in all the primary outcomes of ADHD core symptoms and illness severity compared with TAU/MED group with a medium effect size for the independent raters on the K-SADS total and CGI and slightly less marked findings for the self-reported ADHD symptoms. A significant overall treatment effect was found for the secondary outcome of self-reported depression (small effect size) but, contrary to our hypothesis, this was not quite significant for anxiety and quality of life. As hypothesized, the treatment effect of primary outcomes was maintained at the 3-month follow-up.

Importantly, in contrast to the primary outcome measures, the three secondary measures (anxiety, depression and quality of life) showed a significant improvement over time, all with a medium effect size. This has not been investigated before. One interpretation is that the effect of the CBT treatment is more immediate with regard to the ADHD symptoms and illness severity, whereas symptoms of anxiety and depression and rating of quality of life improve more gradually over time, and may indeed continue to improve. The Emilsson *et al.* (2011) study did not include a measure of quality of life and the current study is the first study in this area to include one at follow-up. An alternative explanation is that the secondary measures effects are programme specific as the *R&R2ADHD* aims to reduce co-morbidity as well as primary symptoms. However, when comparing results from the present study with those reported by Emilsson *et al.* (2011), the effect sizes are generally smaller. This most probably reflects the use of a more sophisticated analysis that better controlled for confounders and thus provided more robust findings. A follow-up of secondary measures should be included in future studies, perhaps using a longer-term follow-up. The findings suggest that focusing exclusively on ADHD measures and symptom severity, like Safren *et al.* (2010), limits the conclusions that can be drawn about other health benefits and improved quality of life. This is particularly important in studies that include participants with a high base level of co-morbid problems. Hirvikoski *et al.* (2011) and Solanto *et al.* (2010) did collect data on co-morbid problems but they only included end-of-treatment data, which showed non-significant results. Ideally, treatment effectiveness should improve over time, or at least be maintained, as participants continue to practise the skills learned during therapy and gradually improve their level of competencies and day-to-day functioning. Further studies need to investigate the mechanisms that facilitate the maintenance and/or changes in outcome over time.

Just over half of the sample completed the group treatment. This is considerably lower than completion rates reported by forensic in-patient samples attending R&R2 (Rees-Jones *et al.* 2012; Cin-Ying Yip *et al*. 2013; Young *et al.* 2013; Waugh *et al.* 2014; Jotangia *et al.* 2015). However, the attrition rate of patients attending community-led treatments has been reported to be high (e.g. Issakidis & Andrews, 2004). Nevertheless, whilst the present study's completion rate is not particularly poor in comparison with other similar community studies, it means that many of the participants missed several of the sessions. In turn this may have adversely influenced the treatment effect, as participants who drop out are unlikely to reach optimal benefit from treatment. Ways for improving treatment completion in community samples need to be considered a priority in future studies.

A large number of participants (in both the CBT/MED and TAU/MED groups) did not attend the sessions to complete their post-treatment assessments. A similar problem with missing data dropout was noted by Emilsson *et al.* (2011) and this was higher than that reported by Safren *et al.* (2010), Solanto *et al.* (2010) and Hirvikoski *et al.* (2011). It is not clear whether this is due to the nature of the participants, the severity of their symptoms, practical considerations such as the Icelandic climate (poor weather and travel conditions), or a reflection of the broader Icelandic community. For example, only 27% of patients attended their follow-up appointment in a large community study evaluating a substance misuse treatment in Reykjavik despite reminders by letter and a subsequent telephone call (Gudjonsson *et al.* 2004). Those patients who failed to attend their follow-up appointment were significantly younger than those who attended, were more antisocial in their personality, and had a higher level of trait anxiety. The current findings confirm the negative relationship between age and attendance at follow-up appointments, but not for ASP traits. Future studies should consider incorporating predictors of missing data into the linear mixed-model analysis in the event of group differences. This is particularly important when comparing participants who drop out of treatment *versus* those who complete all sessions (Everitt & Pickles, 1999).

Strengths of the study were the use of independent raters who were blind to treatment condition and the consistency of these scores with those obtained from the participants’ self-report. This suggests that the participants were not amplifying the benefits of treatment. The study's main limitation is the high dropout rate, which left us with a substantially reduced sample at follow-up. Second, in total there were 10 individual outcome measures (see Table 3), which increase the chance of a type I error although eight out of the 10 variables were significant at ≤0.001, which reduces the likelihood this has occurred. However, the TAU/MED group did not receive any additional control intervention, which may have inflated the treatment effects in the CBT/MED group.

The study adds substantially to knowledge by providing convincing evidence for the effectiveness of *R&R2ADHD* and demonstrates that there are likely to be differential effects over time (end of treatment *versus* follow-up) with regard to primary ADHD outcomes *versus* co-morbid problems. Co-morbid problems are likely to take longer to show positive effect than the core ADHD symptoms. A possible explanation is that the disruptive behaviour commonly arising from ADHD symptoms increases the risk of depression due to the negative reactions of others to their behaviour (Roy, 2014). *R&R2ADHD* treatment improves ADHD symptoms and disruptive behaviour, which over time are likely to reduce co-morbid symptoms due to a more positive reaction from peers, friends and family members. The current time difference found in treatment effects between core ADHD symptoms and co-morbid problems is likely to be related to the different developmental pathways of these conditions.
